# Cross-Sectional Analysis of Sex Differences in Out-of-School Physical Activity Among Elementary School Students

**DOI:** 10.1016/j.focus.2025.100351

**Published:** 2025-04-21

**Authors:** Allison Tep, Paul N. Elish, Peter Boedeker, Hannah K. Behringer, Emilie R. Saksvig, Emily Zheng, Christi M. Kay, Hannah G. Calvert, Adria Meyer, Julie A. Gazmararian

**Affiliations:** 1 Department of Epidemiology, Rollins School of Public Health, Emory University, Atlanta, Georgia; 2 Department of Education, Innovation and Technology, Baylor College of Medicine, Houston, Texas; 3 Center for School and Community Partnerships, College of Education, Boise State University, Boise, Idaho; 4 HealthMPowers, Norcross, Georgia

**Keywords:** Physical activity, accelerometry, school-based health

## Abstract

**Introduction:**

Previous literature has shown differences in physical activity time between boys and girls during the school day. However, these studies did not include physical activity time outside of school. This study aims to explore the differences in physical activity time outside of school, during school, and throughout the full day between fourth grade girls and boys.

**Methods:**

In a large, suburban school district, 386 fourth grade students wore accelerometers both in school and at home for 1 school week. The students also completed an activity diary for one 24-hour period, in which they provided information on physical activities completed before and after school that lasted longer than 5 minutes. Bivariate analyses of demographic characteristics by sex of students were calculated. Bivariate analyses of average moderate-to-vigorous physical activity minutes in school, out of school, and for the full day by demographic characteristics, including sex, were calculated. Random-intercept models estimated associations of interest between physical activity levels and demographic variables.

**Results:**

Girls had significantly fewer minutes of physical activity outside of school, during school, and during the full day than boys. On average, girls accrued 8 less minutes of daily in-school physical activity (19.3 vs 27.1 minutes; *p*<0.001), 7 less minutes of daily out-of-school physical activity (25.1 vs 31.9 minutes; *p*<0.001), and 15 less minutes of full-day physical activity (44.5 vs 59.0 minutes; *p*<0.001) than boys. For both boys and girls, participation in sports was significantly associated with full-day and out-of-school physical activity. Less girls participated in sports than boys (26.4% vs 36.8%).

**Conclusions:**

Differences in elementary-aged physical activity may be due to differences in sex norms and activity outside of school. Girls had less physical activity during all periods of the day than boys and less participation in sports and active commuting. Future research could aim to increase physical activity for elementary-aged girls both in and out of school.

## Highlights

• Physical activity levels in youth may differ by gender.
• Girls appear to be less likely to engage in physical activity compared to boys.
• Physical activity interventions can be developed to address gender disparities.

## INTRODUCTION

Physical activity (PA) during childhood is not only crucial in the prevention of chronic diseases but also in improving overall physical, mental, and social health in children.^1^^,^^2^ Engaging in PA at an early age can reduce the risk factors for chronic diseases, such as heart disease and obesity.^2^^,^^3^ According to the HHS guidelines, individuals aged 6–17 years should complete at least 60 minutes of moderate-to-vigorous PA (MVPA) every day.^2^^,^^3^ The physical activities completed within the 60 minutes should be muscle strengthening, aerobic, and bone strengthening.^2^^,^^3^ Despite the established guidelines and benefits of daily PA engagement, most children aged 6–17 years do not meet the recommended 60 minutes of daily PA.^3^ Furthermore, current literature suggests that disparities in the levels of PA also exist between elementary-aged boys and girls.^4^, ^5^, ^6^

Studies that objectively measured PA have found that girls are less active and spend less time on PA than boys, both in and out of school.^7^^,^^8^ Boys were also more likely to meet fitness standards for aerobic capacity than girls.^9^ Potential explanations could be differences in attitudes that girls have toward PA, such as perceived physical competencies, and participation in organized PA opportunities, such as sports.^10^^,^^11^

Little has been studied on out-of-school–based PA interventions to address differences in PA by sex. Currently, most efforts to increase PA in elementary-aged children have focused on in-school time. Although schools provide an opportune setting for improving PA in children, there is time before school and after school that could be leveraged to ensure that children are meeting the recommended 60 minutes of exercise each day.

Studies of sex differences in PA throughout different periods of the day can inform interventions to promote PA in children. The aims of this study are to expand on current knowledge of gender disparities in PA and to explore out-of-school and full-day PA among girls and boys. In addition, the authors seek to understand how time of day and associated factors, such as active commuting to and from school and participation in sports, may contribute to PA completion by gender.

## METHODS

### Study Population

This study is part of a larger project in which 4,936 Grade 4 students were recruited from 40 elementary schools from a suburban school district in Georgia to participate in the Health Empowers You! Intervention. This intervention aims to enhance PA practices and policies in schools by conducting a baseline assessment on PA practices, training teachers to implement the intervention, providing PA equipment and resources, and offering continuous technical assistance. One PA equipment included accelerometers, which were used to assess the PA duration of students during the school day. Physical activity specialists (PASs) supported the implementation of the intervention by providing technical assistance to the teachers and overseeing the distribution and collection of accelerometers at the beginning and end of the school day. More details regarding school recruitment and intervention activities are provided in a previous study.^12^

For this study, a subset of students was selected to wear accelerometers to measure PA before, during, and after school for a full week of school. The PASs chose 1 classroom from each of the 40 schools with the lowest accelerometer loss rate and the most responsive teacher to participate in this full-day data collection. The school district administration, district IRB, and Emory University IRB approved the study. To be enrolled in the study and for student PA to be measured, parental consent and student assent were obtained.^12^

### Measures

Three data sources were used for this analysis: (1) 24-hour activity diary, (2) accelerometer data, and (3) school district data. The diary was read to students by PASs using a script designed to inquire about different types of activities that were completed throughout the day. For the diaries, students were instructed to select all activities that they completed that lasted 5 minutes or more for one 24-hour period. For before-school activities, students were asked about physical activities completed at home before school in the morning during before-school programs or clubs that were not at their school, their commute to school, and before-school programs or clubs provided at school. For after-school activities, students were similarly asked about physical activities completed during after-school programs or clubs provided at school, their commute home from school, during after-school programs or clubs that were not at the school, and at home after school. Students who were identified as sports participants engaged in a before- or after-school sports program that was either associated with a school or nonschool program. This variable was dichotomized as sports participant and nonsports participant. Students who were identified as active commuters walked, ran, or rode their bike, skateboard, or scooter to and/or from school. This variable was dichotomized as active commuter and nonactive commuter. Out-of-school PA was collected from wake-up time to the start of school and from the end of school to bedtime.

ActiGraph wGT3X-BT 3-axis accelerometers (ActiGraph LLC, Pensacola, FL) were worn by students on the waist from the time they woke up to the time that they went to bed.^12^ The teachers and students were trained by the PASs on how to wear the accelerometers. In addition, the parents of students supported the placement and removal of the accelerometers in the morning and before bedtime, respectively. Data were collected in 15-second epochs and scored by Evenson activity threshold cut points.^12^^,^^13^ Invalid wear time was defined as 60 consecutive minutes of 0 counts, with up to 2 minutes of counts between 0 and 100 allowed.^12^^,^^14^ The intensity and type of PA being collected was the number of minutes of MVPA. Data may be missing owing to reasons such as attendance absences and students forgetting to wear the accelerometer. Participants were excluded from the in-school MVPA analysis if they wore the accelerometer for less than 3 of the 5 days of the school week. Participants were excluded from the out-of-school analysis if they wore the accelerometer for less than 2 of 4 days of the week. Out-of-school data collection only occurred over 4 full days of the school week, because out-of-school MVPA was not collected on Monday morning and Friday evening. These criteria led to the exclusion of 284 students from the 24-hour subset due to insufficient full-day accelerometer data and the inclusion of 386 students. In addition, 15 students completed the 24-hour diary but did not have valid in-school wear time. These 15 students were included in the out-of-school analysis (*n*=386) but are not included in the in-school and full-day analysis (*n*=371).

Student demographic characteristics were obtained using school district data. Demographic characteristics included sex, race, free-reduced lunch (FRL) status, students with disabilities (SWD), and English Language learners (ELLs). Sex was classified as male or female, and race was categorized as Asian, Black, Hispanic, mixed, or White. Students were eligible for FRL if their family household income was <185% of the federal poverty level.^15^

### Statistical Analysis

Of the 670 students who completed activity diaries, 386 students had sufficient accelerometer data for analysis. Descriptive statistics of demographic data were calculated for the students with sufficient accelerometer data (*n*=386). A bivariate analysis of demographic characteristics by sex of students who had sufficient accelerometer data (*n*=386) was also completed and produced chi-square values. In addition, a bivariate analysis of the full-day, in-school, and out-of-school PA of students who had sufficient accelerometer data by demographic characteristics was completed. The reported *t*-test *p*-values for categorical demographic variables with 2 categories were the pooled *p*-values, and the reported general linear model *p*-value for the categorical demographic variable with more than 1 category was the Pr > F value. A general linear model was conducted to analyze the association between sex and MVPA, with school as an effect modifier. Finally, random-intercept multilevel models assessed the association of demographic variables and the PA durations to account for the violation of independence of observation assumption within schools.

## RESULTS

Among the 386 students who had sufficient accelerometer data, a little over half were female and 10.9% Asian, 25.1% Black, 29.3% Hispanic, 4.7% mixed, and 30.1% White (Table 1). Slightly over half of the students did not qualify for FRL, and approximately a tenth was SWD. About 36% of the students were active commuters, and 31% were sports participants.

**Table 1:** Students With Sufficient Accelerometer Data by School Demographics (*n*=386), Grade 4

Demographics	Count (%)
Sex	
Female	201 (52.1)
Male	185 (47.9)
Race	
Asian	42 (10.9)
Black	97 (25.1)
Hispanic	113 (29.3)
Mixed	18 (4.7)
White	116 (30.1)
FRL	
FRL eligible	181 (46.9)
Not FRL eligible	205 (53.1)
ELL^a^	
ELL	77 (22.7)
Not ELL	262 (77.3)
SWD	
SWD	30 (7.8)
Not SWD	356 (92.2)
Active commuting	
Active commuter	140 (36.3)
Not active commuter	246 (63.7)
Sports participation	
Sports participant	121 (31.4)
Not sports participant	265 (68.7)

a A total of 12.2% of students did not have ELL data. FRL, free-reduced lunch; ELL, English language learner; SWD, students with disabilities.

Of the 185 male students, 8.7% were Asian, 29.2% were Black, 27.6% were Hispanic, 6.0% were mixed, and 28.7% were White. Just under half of male students qualified for FRL, a quarter were ELL, and approximately 9% were SWD. Of the 201 female students, 12.9% were Asian, 21.4% were Black, 30.9% were Hispanic, 3.5% were mixed, and 31.3% were White. Similar to the male students, just under half of female students qualified for FRL, 20.9% were ELL, and 6.5% were SWD.

The average time of PA during the full day, in school, and out of school was lower among girls than among boys (Table 2). On average, girls accrued about 15 fewer minutes of full-day PA than boys (44.5 vs 59.0 minutes; *p*<0.001). Girls accrued 8 fewer minutes of in-school PA than boys (19.3 vs 27.1 minutes; *p*<0.001) and accrued approximately 7 less minutes of out-of-school PA than boys (25.1 vs 31.9 minutes, *p*<0.001). Sex was significantly associated with in-school, out-of-school, and full-day PA (*p*<0.001) (Table 2). Sports participation outside of school was significantly associated with full-day and out-of-school PA (*p*<0.001), whereas active commuting was not significantly associated with any time period (Table 2). Sports participants had higher out-of-school PA than nonsports participants (35 vs 25.3 minutes, *p*<0.001) and higher full-day PA than nonsport participants (59.1 vs 48.3 minutes; *p*<0.001). Significantly fewer girls participated in sports (26.4% vs 36.8%; *χ*^2^=0.0280) than boys (Table 3). Sex differences in full-day MVPA averages (*p*=0.3227), out-of-school MVPA averages (*p*=0.0829), and in-school MVPA averages (*p*=0.2115) did not vary by school. Results from multilevel models showed that the time spent completing MVPA was less for girls than for boys for full-day, in-school, and out-of-school MVPA (ß_full-day_= −11.89, ß_in-school_= −7.205, ß_out-of-school_= −5.025, *p*=0) (Table 4).

**Table 2:** Bivariate Analysis of PA Measures by Characteristics of Students Who Wore Accelerometers

PA outcome	Full-day PA (minutes) (*n*=371)		In-school PA (minutes) (*n*=371)			Out-of-school PA (minutes) (*n*=386)	
	Mean (SD)	*p*-value	Mean (SD)		*p*-value	Mean (SD)	*p*-value
Sex							
Female	44.5 (17.5)	**<0.001**	19.3 (7.6)		**<0.001**	25.1 (13.9)	**<0.001**
Male	59.0 (20.8)		27.1 (10.6)			31.9 (15.2)	
Race		**<0.001**			**<0.001**		0.0017
Asian	40.1 (14.5)		18.2 (6.5)			21.3 (11.3)	
Black	58.5 (20.5)		26.7 (11.2)			31.2 (16.2)	
Hispanic	49.6 (22.5)		22.5 (10.5)			27.3 (15.1)	
Mixed	62.8 (25.0)		27.9 (12.2)			34.9 (16.0)	
White	49.9 (16.5)		21.6 (7.6)			28.5 (13.6)	
FRL status		0.4419			0.7891		0.5017
FRL	50.7 (21.5)		23.0 (10.7)			27.8 (14.7)	
Not FRL	52.3 (19.5)		23.2 (9.3)			28.8 (15.1)	
ELL^a^		0.8151			0.4026		0.8426
ELL	51.8 (24.0)		22.5 (9.9)			29.1 (17.2)	
Not ELL	52.4 (19.5)		23.6 (10.0)			28.7 (14.5)	
SWD		0.4447			0.0337		0.7356
SWD	54.4 (27.4)		27.0 (11.0)			27.5 (17.9)	
Not SWD	51.3 (19.8)		22.8 (9.8)			28.4 (14.6)	
Active commuting		0.5035			0.0390		0.6326
Active	52.5 (19.5)		24.5 (10.7)			27.9 (13.4)	
Not active	51.0 (21.0)		22.3 (9.5)			28.6 (15.7)	
Sports participation		**<0.001**			0.2842		**<**0.001
Participant	59.1 (21.8)		24.0 (10.7)			35.0 (15.6)	
Not participant	48.3 (19.0)		22.7 (9.6)			25.3 (13.5)	

*Note:* Boldface denotes statistical significance (*p*<0.05).

a Approximately 12% of students did not have ELL data for each PA measure. ELL, English language learner; FRL, free-reduced lunch; PA, physical activity; SWD, students with disabilities.

**Table 3:** Bivariate Analysis of Demographic Characteristics by Sex of Students Who Wore Accelerometers (*n*=386)

**Variable**	**Sex**				
	**Male (*n*=185) (*n*)**	**%**	**Female (*n*=201) (*n*)**	**%**	**Chi-square value**
Race					0.2148
Asian	16	8.7	26	12.9	
Black	54	29.2	43	21.4	
Hispanic	51	27.6	62	30.9	
Mixed	11	6.0	7	3.5	
White	53	28.7	63	31.3	
FRL					0.7211
FRL eligible	85	46.0	96	47.8	
Not FRL eligible	100	54	105	52.2	
ELL^a^					0.4058
ELL	40	24.7	37	20.9	
Not ELL	122	75.3	140	79.1	
SWD					0.3184
SWD	17	9.2	13	6.5	
Not SWD	168	90.8	188	93.5	
Active commuting					0.2989
Active commuter	72	38.9	68	33.8	
Not active commuter	113	61.1	133	66.2	
Sports participation					0.0280
Sports participant	68	36.8	53	26.4	
Not sports participant	117	63.2	148	73.6	

a Approximately 12% of students did not have ELL data. ELL, English language learner; FRL, free-reduced lunch; PA, moderate to vigorous PA; SWD, students with disabilities.

**Table 4:** Associations Between PA^1^ and Demographic Characteristics (*n*=386)

	Full-day PA^1^		In-school PA^1^		Out-of-school PA^1^	
PA^1^ outcome	Coefficient (SE)	*p*-value	Coefficient (SE)	*p*-value	Coefficient (SE)	*p*-value
Sex						
Male^2^	ref		ref		ref	
Female	−11.89 (1.912)	0	−7.205 (0.848)	0	−5.025 (1.425)	0
Race						
White^2^	ref		ref		ref	
Asian	−11.157 (3.688)	0.002	−3.347 (1.622)	0.039	−8.323 (2.747)	0.002
Black	6.985 (2.711)	0.01	3.886 (1.219)	0.001	2.237 (2)	0.263
Hispanic	−0.45 (3.097)	0.884	0.92 (1.384)	0.506	−1.363 (2.283)	0.55
Mixed	11.856 (4.633)	0.011	4.064 (2.062)	0.049	7.197 (3.513)	0.041
FRL						
No FRL^a^	ref		ref		ref	
FRL	−2.592 (2.32)	0.264	−1.518 (1.059)	0.152	−0.965 (1.686)	0.567
ELL						
No ELL^a^	ref		ref		ref	
ELL	3.842 (2.761)	0.164	−0.018 (1.225)	0.988	2.878 (2.061)	0.163
SWD						
No SWD^a^	ref		ref		ref	
SWD	0.878 (3.549)	0.805	2.316 (1.564)	0.139	−2.005 (2.59)	0.439
Active commuting						
Not active commuter^a^	ref		ref		ref	
Active commuter	2.313 (2.096)	0.27	1.906 (0.969)	0.049	0.109 (1.5)	0.942
Sports participation						
Not sports participant^a^	ref		ref		ref	
Sports participant	9.152 (2.072)	0	0.26 (0.919)	0.777	9.285 (1.533)	0

a For analysis, reference group comprised White male, not eligible for free or reduced-price meals, not classified as ELL, not classified as SWD, not an active commuter, and not a sports participant. ELL, English language learner; FRL, free-reduced lunch; PA, physical activity; SWD, students with disabilities.

## DISCUSSION

The results suggest that differences in out-of-school; in-school; and, subsequently, full-day average PA may be present among fourth-grade–aged boys and girls. Boys may be receiving more average minutes of PA during the day than girls. The results indicate that for PA outside of school, sports participation before or after school may lead to higher levels of PA outside of school than active commuting. Because sports participation was lower in girls than in boys (26.4% vs 36.8%), this may contribute to the lower number of average minutes of PA completed by girls. On the basis of these results, one strategy with which to increase PA levels among girls could be through the enrollment in before- or after-school programs or clubs that would allow girls to participate in sports. These opportunities could be provided through schools or outside of school.

A few studies have found that children’s engagement in PA decreases as they enter adolescence.^6^^,^^7^ This finding adds to the importance of addressing low levels of PA in girls during elementary-aged years to influence trends of PA levels in adolescence. In addition, current interventions that intend to increase adolescent PA are Girls on the Run, which aims to increase motor skills and PA for girls aged 8–14 years, and Girls Empowering Movement in Georgia, which aims to increase adolescent PA outside of school.^16^^,^^17^

Strengths of the study include the diversity of the sample and the objective measurement of PA.^12^ The students who completed the activity diaries were almost equal proportions of Black, Hispanic, and White students, and almost half of students were FRL eligible and female. The activity diary data, combined with the accelerometer data, provide a wide variety of PA types to be studied. The diary inquired about traditional forms of PA, such as sports; walking; running; exercising; and riding different modes of transportation, such as a bike or scooter. The diary also inquired about nontraditional forms of PA, such as indoor and outdoor household chores.

### Limitations

The validity criteria for accelerometer wear time led to the exclusion of 284 participants from the larger subset of students who completed an activity diary (*n*=670). More girls (*n*=201, 52.1%) and fewer boys (*n*=185, 47.9%) had valid accelerometer data. In addition, the study population has a lack of generalizability owing to the sample being limited to suburban schools, fourth graders, and the nonrandom selection of classrooms to participate in the 24-hour subset. These limitations may cause the findings to not be applicable to other elementary-grade levels.

## CONCLUSIONS

The study analysis indicated that girls consistently engage in less PA than boys during all time periods of the day. Although all PA time periods were significantly associated with sex, this association was not as consistent across other demographic variables. Future research opportunities could further explore the factors, such as sex and participation in sports programs and clubs, that contribute to out-of-school PA types and completion. This study's results can inform the development of future interventions that encourage elementary-aged girls to be more physically active outside of school.

## CRediT authorship contribution statement

**Allison Tep:** Formal analysis, Writing – review & editing. **Paul N. Elish:** Formal analysis, Data curation, Writing – review & editing. **Peter Boedeker:** Data curation, Formal analysis, Writing – review & editing. **Hannah K. Behringer:** Writing – original draft, Writing – review & editing. **Emilie R. Saksvig:** Writing – original draft, Writing – review & editing. **Emily Zheng:** Writing – review & editing. **Christi M. Kay:** Investigation, Funding acquisition, Writing – review & editing. **Hannah G. Calvert:** Investigation, Data curation, Writing – review & editing. **Adria Meyer:** Investigation, Writing – review & editing. **Julie A. Gazmararian:** Supervision, Data curation, Resources, Visualization, Project administration, Funding acquisition, Writing – review & editing.
